# Intelligent Detection of Steel Defects Based on Improved Split Attention Networks

**DOI:** 10.3389/fbioe.2021.810876

**Published:** 2022-01-13

**Authors:** Zhiqiang Hao, Zhigang Wang, Dongxu Bai, Bo Tao, Xiliang Tong, Baojia Chen

**Affiliations:** ^1^ Key Laboratory of Metallurgical Equipment and Control Technology of Ministry of Education, Wuhan University of Science and Technology, Wuhan, China; ^2^ Hubei Key Laboratory of Mechanical Transmission and Manufacturing Engineering, Wuhan University of Science and Technology, Wuhan, China; ^3^ Precision Manufacturing Research Institute, Wuhan University of Science and Technology, Wuhan, China; ^4^ Research Center for Biomimetic Robot and Intelligent Measurement and Control, Wuhan University of Science and Technology, Wuhan, China; ^5^ Hubei Key Laboratory of Hydroelectric Machinery Design and Maintenance, Three Gorges University, Yichang, China

**Keywords:** defect detection, target identification, attention mechanism, feature extraction and fusion, split attention networks

## Abstract

The intelligent monitoring and diagnosis of steel defects plays an important role in improving steel quality, production efficiency, and associated smart manufacturing. The application of the bio-inspired algorithms to mechanical engineering problems is of great significance. The split attention network is an improvement of the residual network, and it is an improvement of the visual attention mechanism in the bionic algorithm. In this paper, based on the feature pyramid network and split attention network, the network is improved and optimised in terms of data enhancement, multi-scale feature fusion and network structure optimisation. The DF-ResNeSt50 network model is proposed, which introduces a simple modularized split attention block, which can improve the attention mechanism of cross-feature graph groups. Finally, experimental validation proves that the proposed network model has good performance and application prospects in the intelligent detection of steel defects.

## 1. Introduction

The application of Bio-inspired computation and artificial intelligence technology is gradually taking an important position in the field of mechanical engineering. More specifically, bio-inspired algorithms can replace humans to a certain extent, through training and learning to complete the tedious task of detecting steel surface defects (Chen et al., 2021a; Yang et al., 2021; Yun et al., 2021, 2022). Research on steel plate defect detection based on visual attention mechanisms and bionic algorithms will help the steel industry move towards intelligence and information.

Currently, the detection of steel plate defects is still dominated by manual inspection, i.e., manual visual inspection or random sampling of products (Tang et al., 2017; Yu et al., 2019, Yu et al., 2020; Jawahar et al., 2020; Tian et al., 2020). However, manual inspection has problems such as strong subjectivity, limited vision and low efficiency, which to a certain extent restrict the intelligent and efficient production in the steel industry (Sun et al., 2020a; Sun et al., 2020b; Jiang et al., 2021b; Zhao et al., 2021). Meanwhile, eddy current inspection, infrared inspection, leakage magnetic inspection, laser scanning, and machine vision have facilitated the equipment-based inspection of steel, but there are still problems such as low speed and accuracy of defect detection.

**FIGURE 1:**
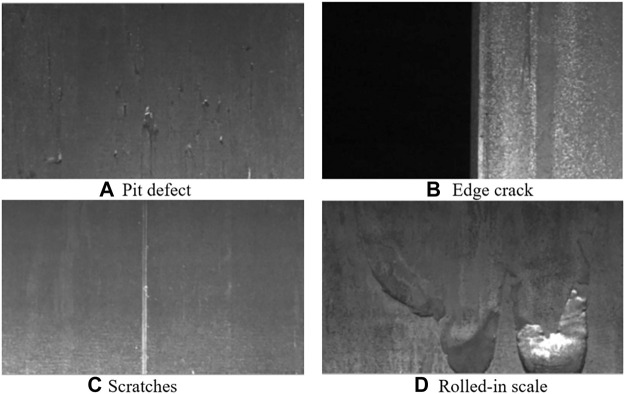
Four typical steel plate defects. **(A)** Pit defect, **(B)** Edge crack, **(C)** Scratches, **(D)** Rolled-in scale.

For steel plate defects, the types of defects are complex and diverse, and there are many influencing factors, and the shape of defects will continue to change with factors such as process and environment, which adds many challenges to steel defect detection (Li et al., 2019a; Hao et al., 2021). Figure 1 shows the four typical steel plate defects: (a) Pit defect, (b) Edge crack, (c) Scratches, (d) Rolled-in scale.

The key contributions of this work are:

1) The steel plate defect dataset is masked using Run-Length encoding, and the defect detection model is segmented using multi-scale feature fusion.
2) Based on the visual attention mechanism in the bio-inspired algorithms, combined with the feature pyramid network, on the basis of the residual network, a simple modular split-attention block is added, and the DF-ResNeSt50 network is proposed.
3) DF-ResNeSt50 network adopts radix-major to realize the block, the block is set to Cardinality = 2, Radix = 4, Width of bottleneck = 40. The proposed DF-ResNeSt50 algorithm is analyzed and compared with other classical algorithms. After experimental comparison, the network has better steel defect detection performance and detection efficiency.

The rest of this paper is organized as follows: Section 2 discusses the related work of steel plate surface defect detection in recent years. Section 3 briefly analyzes the data set, and proposes to use Run-Length encoding to compress the data and perform data preprocessing. In addition, an improved split-attention network based on the visual attention mechanism in bionic computing is proposed for residual networks and feature pyramid networks. Before network training, use mIou, Dice and other related indicators to monitor, and use Adam to dynamically adjust and optimize the learning rate. Section 4 compares and trains the proposed DF-ResNeSt50 network model after setting up the experimental environment and hyperparameters, and compared with other network models. Section 5 concludes the paper with summary and future research directions.

## 2. Related Work

In the surface defect detection system, image processing and analysis algorithms are important content. The usual process includes image preprocessing, target area segmentation, feature extraction and selection, and defect recognition and classification (Weng et al., 2021). As the requirements for the surface quality of steel plates become higher and higher, the requirements for real-time detection and recognition accuracy are also higher and higher. A large number of algorithms appear in each processing flow, and these algorithms have their own advantages and disadvantages and their scope of adaptation (Doulgkeroglou et al., 2020; Luo et al., 2020).

Compared with traditional manual features, the biggest advantage of deep learning is that it can automatically learn the performance of complex high-level features in the data, reducing the complexity of manual feature design. In recent years, deep learning has been successfully applied to speech recognition, image recognition, image segmentation, defect detection and other fields (Huang et al., 2020; Li et al., 2020).

In the application of deep learning, Çelik et al. (2014) designed a defect detection method that applies wavelet transform ideas to neural networks, and experiments have proved that this method has excellent defect detection performance (Long et al., 2015). Li et al. (2017) designed a stack noise reduction autoencoder based on the Fisher criterion and built a defect detection model based on this, which can improve the recognition rate of defect types to a certain extent (Xiao et al., 2021). Gu et al. (2019) established a detection and recognition model for cold-rolled steel sheet surface defects based on the deep learning target detection algorithm Faster R-CNN (Jiang et al., 2021b). The accuracy of the model on the verification set reached an average of 93%. He et al. (2020) introduced a transfer learning method, using feature extraction networks trained on large-scale data sets to greatly improve training efficiency.

At present, deep learning algorithms are rarely applied to the detection of steel surface defects. The above-mentioned intelligent detection method for steel plate defects based on deep learning still has problems such as low classification rate and low accuracy of defect target detection. In a complex environment, the stability and robustness of the neural network detection system is difficult to guarantee (Chen et al., 2021b; Duan et al., 2021).

With the widespread use of deep learning, convolutional neural networks will have better defect feature recognition and detection capabilities. In this paper, convolutional neural networks are used to intelligently detect defects in steel plates to improve the automation and intelligence of defect detection in the steel industry.

## 3. Data Analysis and Network Design

### 3.1 Data Analysis and Processing

#### 3.1.1 Dataset

The steel plate defect dataset studied in this paper comes from the competition platform kaggle and the Russian steel giant Severstal. The data set contains 12,568 pieces of test set data and 1801 pieces of training set data. The vertical and horizontal resolutions of the picture are 256 and 1600 respectively.

**FIGURE 2:**
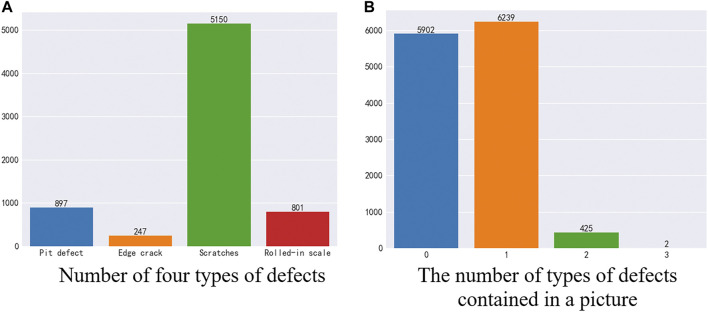
Training set analysis. **(A)** Number of four types of defects **(B)** The number of types of defects contained in a picture.

As shown in Figure 2, in the training data, there are 5902 images without defects and 6666 images with defects. In a defective image, there are four types of defects, and the numbers of the four types of defects are not equal. Among them, 897 were pit defects, 247 were edge crack defects, 5,150 were scratch defects, and 801 were oxide scale defects. And, there are 6239 images containing 1 type of defect, 425 images containing two types of defects, 2 images containing three types of defects, and no images containing four types of defects.

**FIGURE 3:**
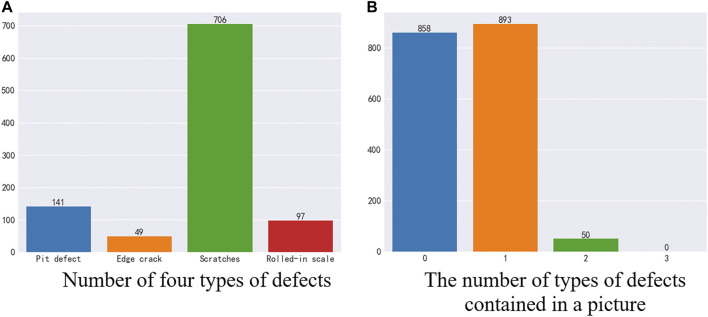
Test set analysis. **(A)** Number of four types of defects **(B)** The number of types of defects contained in a picture.

As shown in Figure 3, in the test set data, there are 858 images without defects and 943 images with defects. In a defective image, there are four types of defects, and the numbers of the four types of defects are not equal. Among them, 141 were pit defects, 49 were edge crack defects, 706 were scratch defects, and 97 were oxide scale defects. In each image, there are 893 images containing one defect, 50 images containing two types of defects, and no images containing three or four defects at the same time. As shown in Figure 3.

From the analysis of the data set, the number of defective and non-defective images is roughly the same. The number of different types of defects is unbalanced, but the corresponding proportions of defects in the training set and the test set are the same. And most images have no defects or only contain one type of defect. This brings great difficulties and challenges to neural network construction and network training.

#### 3.1.2 Run-Length Encoding

Because the defect image has a resolution of 256 × 1600, the size is too large to limit the computing power and neural network model, and it also has a great impact on the detection of small defects. This paper uses Run-Length Encoding (RLE) algorithm to compress the data.

RLE is a simple lossless compression method, which is characterized by very fast compression and decompression (Li et al., 2021). This method uses repeated bytes and the number of repetitions to simply describe the repeated bytes, that is, a series of consecutive identical data is converted into a specific format to achieve the purpose of compression.

Meanwhile, in order to reduce the amount of calculation and compress the deep learning model, this paper uses the parameter quantization method to train the network, and uses FP32 and uint8 mixed training to reduce the memory usage and training time of the model.

#### 3.1.3 Data Preprocessing

Data preprocessing is an essential step before neural network training and testing. The quality of preprocessing will directly determine the training results. Based on pytorch, this paper uses torchvision graphics library to process data sets.

The data were processed and enhanced by ColorJitter (modifying brightness, contrast and saturation), RandomVerticalFlip (flipping vertically around *X* axis according to probability), RandomHorizontalFlip (flipping horizontally around *Y* axis according to probability). Finally, the data were regularized [mean = (0.485, 0.456, 0.406), std = (0.229, 0.224, 0.225)] and normalized.

The data preprocessing settings are shown in Table 1.

**TABLE 1:** Data enhancement method.

Image enhancement method	Probability
ColorJitter	0.5
RandomVerticalFlip	0.5
RandomHorizontalFlip	0.5
Normalize	1

In this section, the necessary analysis of the data set is carried out, and the data set is coded, decoded and processed to provide help for network training.

### 3.2 Model Evaluation Indicators and Parameter Settings

#### 3.2.1 Evaluation Index

In the field of semantic segmentation, IoU(Intersection over Union), mIoU (mean Intersection over Union) and Dice are important evaluation indicators to measure the accuracy of image segmentation.

mIoU, i.e., calculating the IoU values on each category and then averaging them. It is calculated as TP (number of true samples)/[TP (number of true samples) + FN(number of false negative samples) + FN(number of false positive samples) numbers] (He et al., 2019; Cheng et al., 2020, 2021).

$$MIoU=\frac{1}{k+1}\sum_{i=0}^{k}\frac{p_{ii}}{\sum_{j=0}^{k}p_{ij}+\sum_{j=0}^{k}p_{ji}-p_{ii}}$$ (1)

Equivalent to:

$$MIoU=\frac{1}{k+1}\sum_{i=0}^{k}\frac{TP}{FN+FP+TP}$$ (2)

In which, *i* represents the true value, *j* represents the predicted value, represents the prediction of the i-type pixel as the j-type pixel, and *k* is the total number of categories. *TP* (True Positive) means that the prediction is correct and the prediction result is correct. *FP* (False Positive) means that the prediction is wrong and the prediction result is correct. *FN* (False Negative) means that the prediction is correct, but the prediction result is wrong.

$$Dice\left(X,Y\right)=\frac{2\left|X\cap Y\right|}{X+Y}$$ (3)

Where, *Dice* is a common indicator in medical images. *X* represents the real result, *Y* represents the predicted result, and X∩Y represents the correct result of the prediction.

#### 3.2.2 Weighted Loss Function

In deep neural networks, the loss function is used as an important indicator to evaluate the accuracy of the model, which provides a reference for the network model to approach the high-precision direction (Zhang H. et al., 2021). Decreasing the Loss value of the network model can make the model more and more accurate and improve the robustness of the model. Common loss functions include logarithmic loss function, mean square error loss function (MSE), cross entropy loss function, and exponential loss function.

In the multi-label classification problem, the binary cross entropy loss function (BCE Loss) is the most common. BCE Loss is defined as follows:

$$L_{BCE}\left(\hat{y},y\right)=-\frac{1}{n}\sum_{i=1}^{n}\left(y_{i}\log{\hat{y}}_{i}+\left(1-y_{i}\right)\log\left(1-{\hat{y}}_{i}\right)\right)$$ (4)

In which, *n* represents the total number of samples in the training set, $y_{i}$ represents the true label of the *i*th sample, and ${\hat{y}}_{i}$ represents the model prediction value of the *i*th sample.

Sigmoid is a differentiable bounded function with non-negative derivatives at every point. It is often used in binary classification problems, as well as the activation function of neural networks (Ma et al., 2020), that is, to convert linear input into non-linear output.

$$S\left(x\right)=\frac{1}{1+e^{-x}}$$ (5)

The form of the Sigmoid function is shown in Eq. 5. When x→∞ , S(x)→1 ; when x→−∞ , S(x)→0 .

This paper will use BCEWithLogitsLoss as the loss function for the intelligent detection of steel defects. BCEWithLogitsLoss combines the BCELoss and Sigmoid functions into one category, which is numerically more stable than using ordinary BCELoss and Sigmoid.

#### 3.2.3 Learning Rate Adjustment and Its Optimizer

If the initial learning rate is too large, it will cause oscillation; the initial learning rate is too small, resulting in slow convergence; the later learning rate is too large, it will cause overfitting. Therefore, in the training process, a dynamically changing learning rate is generally set according to the number of training rounds. The ideal strategy is to start with a large learning rate and gradually decay.

In this paper, the ReduceLROnPlateau method is used to dynamically update the learning rate, which is based on the number of epoch training times and some measurement values (loss, accaurcy, etc.) to dynamically decrease the learning rate.

Adaptive Moment Estimation (Adam) is an optimizer that converges quickly and is often used. Adam uses the first-order moment estimation and the second-order moment estimation of the gradient to dynamically adjust the learning rate (Liao et al., 2020; Liao et al., 2021; Jiang et al., 2019a). It is an optimisation method of adaptive learning rate. This paper uses Adam optimization method to continuously optimize the learning rate.

### 3.3 Defect Detection Network Structure

#### 3.3.1 Backbone

**FIGURE 4:**
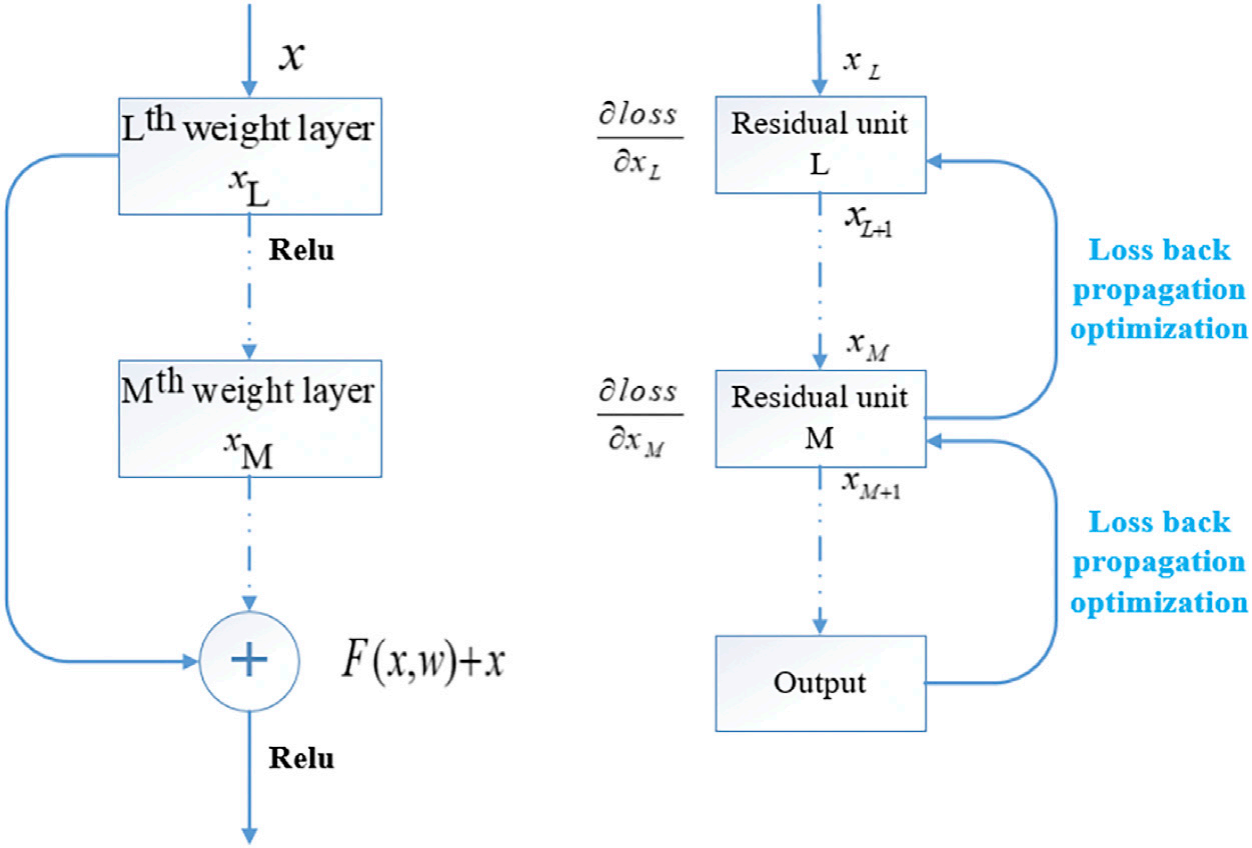
Residual module structure of ResNet.

He and others proposed residual network (ResNet; He et al., 2016), which has become one of the most widely used CNN basic feature extraction networks in the field of computer vision by introducing the concept of residual learning into CNN (He et al., 2014). The residual block introduced in ResNet solves the problem of network performance degradation caused by gradient dispersion in the process of continuous deepening of the network. The residual module structure of ResNet is shown in Figure 4.

During forward propagation, due to the existence of short-circuit connections, the deep and shallow features satisfy the relationship:

$$x_{L+1}=x_{L}+F\left(x_{L},W_{L}\right)$$ (6)

$$x_{M}=x_{L}+\sum_{i=L}^{M-1}F\left(x_{i},W_{i}\right)$$ (7)

In which, $x_{L}$ and $x_{L+1}$ represent the input and output features of the *L*th layer residual unit, $F\left(x_{L},W_{L}\right)$ represents the residual mapping learned by the network, and $x_{M}$ represents the input feature of the *M*th layer residual unit. Based on the chain derivation rule, the gradient during backpropagation is:

$$\frac{\partial loss}{\partial x_{L}}=\frac{\partial loss}{\partial x_{M}}\cdot \frac{\partial x_{M}}{\partial x_{L}}=\frac{\partial loss}{\partial x_{M}}\cdot \left(1+\frac{\partial }{\partial x_{L}}\sum_{i=L}^{M-1}F\left(x_{i},W_{i}\right)\right)$$ (8)

It can be seen that the residual module establishes a short-circuit connection between the input and output of the module through identity mapping, so that the gradient can be maintained during back propagation and the gradient dispersion phenomenon can be alleviated (Li et al., 2019b; Tan et al., 2020).

Meanwhile, ResNet uses a bottleneck structure to replace the original two 3 × 3 convolutions of the residual module, with significantly fewer parameters in the same input dimension. By stacking the basic units of the residual module, the depth of the network can break through the original limit and reach hundreds of layers.

Good performance of ResNet on image recognition and localization tasks showed that characterisation depth is of central importance for many visual recognition tasks. In the following years, excellent feature extraction networks such as ResNeXt, DenseNet, RegNet, SEnet, SKNet, etc (Xie et al., 2017; Liu X. et al., 2021; Liu et al., 2021b; Liu et al., 2021c; Liu et al., 2021d; Sun et al., 2020c; Sun et al., 2020d). were successively proposed, constantly refreshing the accuracy rate of tasks such as image classification. However, most of these detection algorithms have been studied based on ResNet for improvement.

#### 3.3.2 Architectures

Target detection tasks and semantic segmentation tasks often need to detect small targets, and the data set in this article has small defect targets that need to be detected. However, in the deep learning model, after many layers of convolution, the characteristics of small targets will become fewer and smaller.

Feature Pyramid Networks (FPN) was proposed by Lin Tsung-Yi and others in 2017 (Lin et al., 2017). FPN introduces multi-scale in the feature pyramid network and improves on the basis of the SSD multi-layer branching method (Bai et al., 2021; Cui et al., 2021). Similar to the TDM (Top-Down Modulation) method, FPN is a top-down feature fusion method.

Feature pyramid networks is a multi-scale target detection algorithm, that is, there is more than one feature prediction layer. Although some algorithms also use multi-scale feature fusion for target detection, they often only use the features of one scale obtained after fusion. Although this approach can combine the semantic information of the top-level features and the detailed information of the bottom-level features, it will cause some deviations in the process of feature deconvolution, and only using the features obtained after fusion for prediction will adversely affect the detection accuracy (Huang et al., 2019; Jiang et al., 2019b). Starting from the above-mentioned problems, the FPN method can predict on multiple fusion features of different scales to maximize the detection accuracy.

**FIGURE 5:**
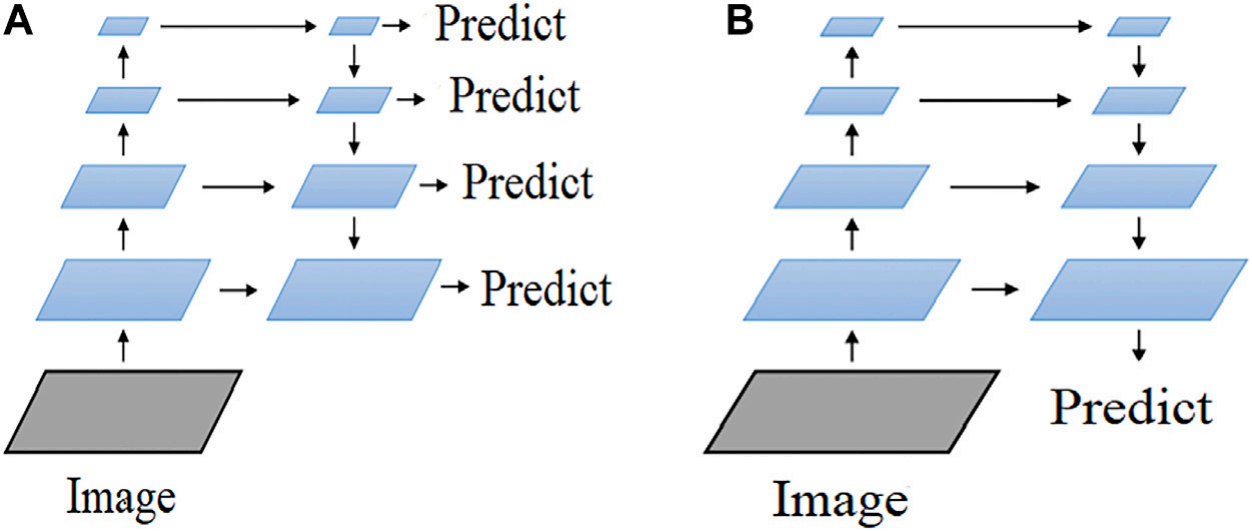
FPN architecture.

As shown in Figure 5, the branches corresponding to the left half of the two networks in Figure 5 is the pre-trained network. Since the whole flow is bottom-up, it is called a bottom-up network. The entire flow of the branches corresponding to the right half of the two networks is top-down. The so-called top-down network is the core part of the FPN. The diagram shows (a) predictions for each layer of the network, and (b) predictions for the final layer after fusing the multi-scale features. In general, the (a) graph structure is widely used for target detection and semantic segmentation, while the (b) structure is more often used for semantic segmentation (Sun et al., 2021; Tao et al., 2022).

FPN uses a multi-feature fusion approach to improve the accuracy of the model. In this paper, FPN is applied to ResNeSt and improved and optimised accordingly to achieve better segmentation of steel surface defects.

### 3.4 DF-ResNeSt50

In 2020, Split-Attention Networks (ResNeSt) was proposed (Zhang M. et al., 2021). ResNeSt introduces the Split-Attention block, which consists of a feature map group and split attention operation.

**FIGURE 6:**
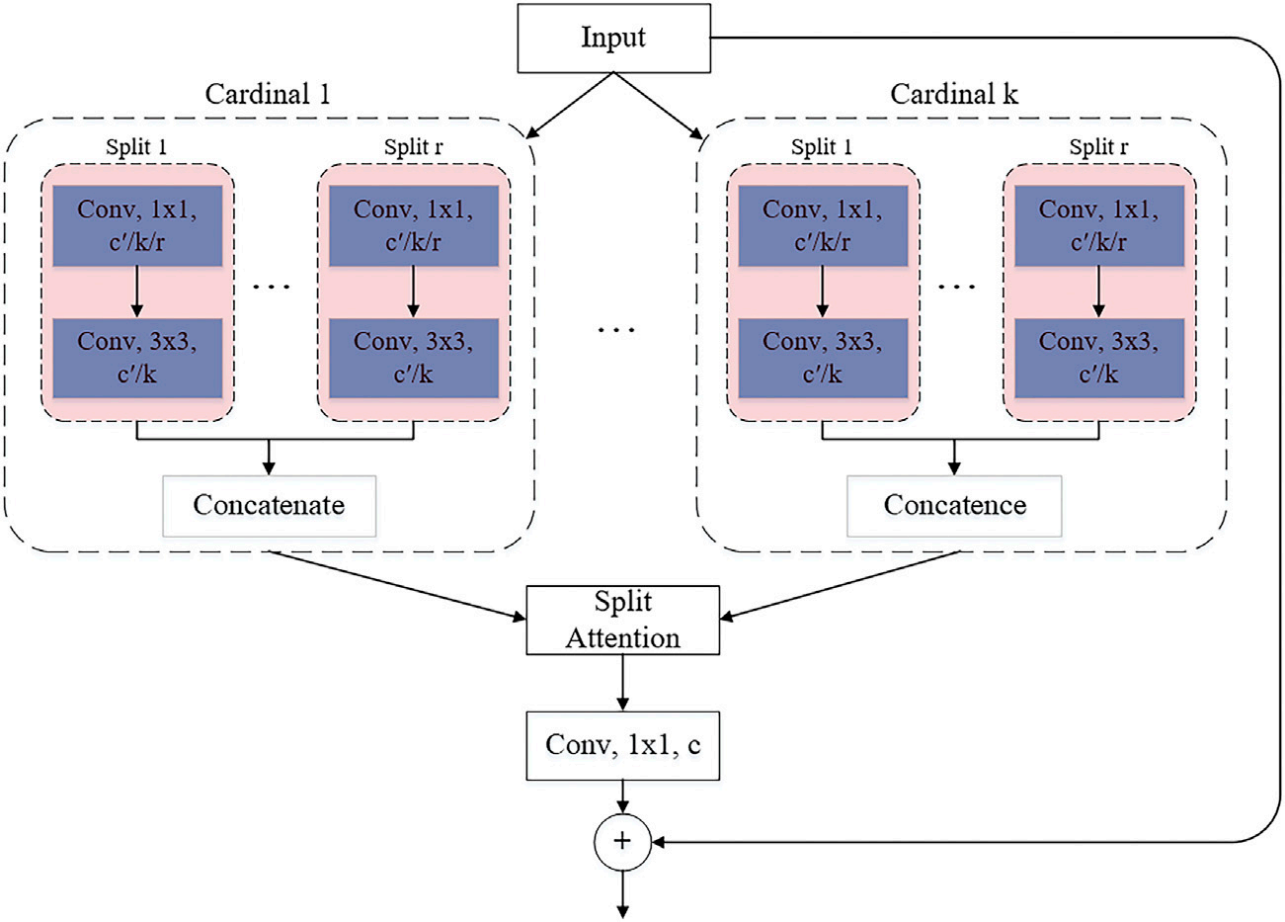
ResNeSt block.

The number of feature map groups is given by the cardinal hyperparameter **k**. The network refers to the generated feature map group as a cardinality array. And introduce a new base number called hyperparameter **r**, which represents the number of splits in the cardinal array. The total number of feature groups is **G = kr**, as shown in Figure 6.

The layout of Figure 6 is a cardinality master implementation, in which feature map groups with the same cardinality index are physically adjacent to each other. The cardinal implementation is simple and intuitive, but it is difficult to use standard operators for modularization and acceleration. For this reason, an equivalent base-first implementation has been introduced.

The combined representation of each cardinal group can be obtained by summing and fusing the elements across multiple splits. The representation of the *k*th cardinal group is:

$${\hat{U}}^{k}=\sum_{j=\left(k-1\right)r+1}^{kr}U_{j}$$ (9)

Here the *c*th component is calculated as:

$$s_{c}^{k}=\frac{1}{H\times W}\sum_{i=1}^{H}\sum_{j=1}^{W}{\hat{U}}_{c}^{k}\left(i,j\right)$$ (10)

In which, each feature map channel is generated using a combination of weighted splits. H, W and C are the size of the block output feature map. The Eq. 10 for the *c*th channel is:

$$v_{c}^{k}=\sum_{i=1}^{r}a_{i}^{k}\left(c\right)U_{r\left(k-1\right)+i}$$ (11)

Where $a_{i}^{k}\left(c\right)$ denotes a (soft) assignment weight given by:

$$a_{i}^{k}\left(c\right)=\left\{ \begin{array}{l} \frac{\exp\left(\vartheta_{i}^{c}\left(s^{k}\right)\right)}{\sum_{j=1}^{r}\exp\left(\vartheta_{j}^{c}\left(s^{k}\right)\right)}\;if\;r>1\\ \frac{1}{1+\exp\left(-\vartheta_{i}^{c}\left(s^{k}\right)\right)}\;if\;r=1 \end{array}\right.$$ (12)

**FIGURE 7:**
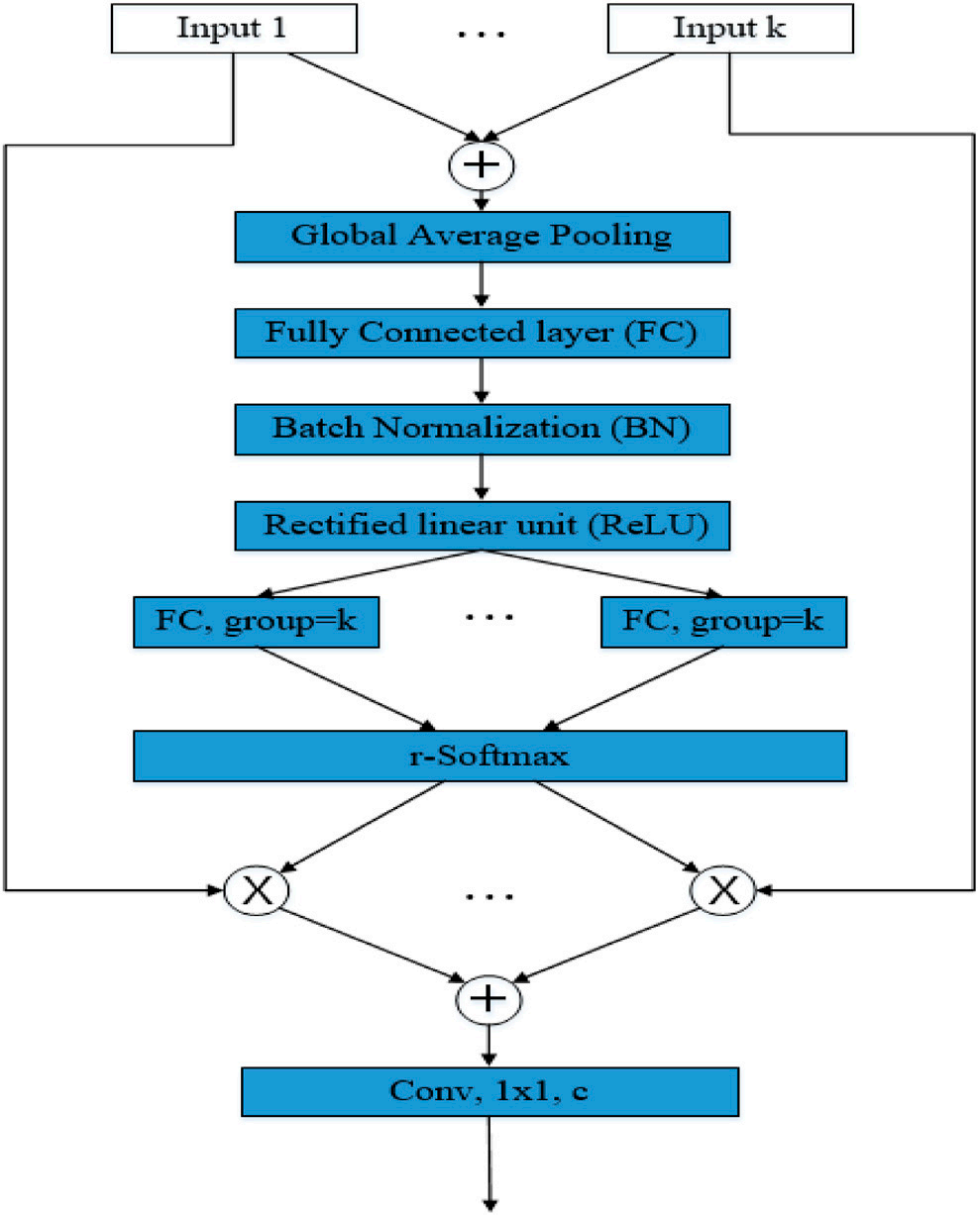
Split attention module.

Based on the weights of the global contextual information, mapping $\vartheta_{i}^{c}$ denotes $s^{k}$ , representing the weight of the split attention of the *c*th channel. As shown in Eq. 12 and Figure 7, when r = 1, r-Softmax is sigmoid, and when r > 1, r-Softmax is softmax.

Figure 7 outlines the split attention block in a radix-major layout. The input feature map is first divided into **kr** groups, where each group has a cardinality index and a radix index. In this layout, groups with the same cardinality index are adjacent to each other. The divisions of the different groups are then added together to combine feature maps with the same cardinality index but different cardinality indexes. The global pooling layer aggregates in the spatial dimension, keeping the channel dimensions separate, and performs global pooling on each individual cardinal array. Finally, two consecutive fully-connected (FC) layers with a group size equal to **k** are added after the pooling layer to predict the attention weights for each segmentation. The two FC layers are activated using BN and ReLU in between, and the use of grouped FC layers makes it identical to apply each pair of FCs to the top of each cardinal array separately.

With this implementation, the first 1 × 1 convolutional layer can be unified into a single layer, and the 3 × 3 convolutional layer can be implemented with a single grouped convolution of the **kr** number of groups. The Split attention module can therefore be modularised using standard operators to improve network efficiency easily and quickly.

In this paper, the FPN and ResNeSt are modified and fused to complete the network construction, and named DF-ResNeSt50. DF-ResNeSt50 uses Cardinality(**k**) = 2, Radix(**r**) = 4 and width of bottleneck = 40.

State-of-the-art performance can be achieved on multiple tasks using the improved ResNeSt backbone model, namely: image classification, target detection, instance segmentation and semantic segmentation (Huang et al., 2021; Jiang et al., 2019c). ResNeSt outperforms all existing variants of ResNet and has the same computational efficiency. Therefore, this paper uses DF-ResNeSt50 for network training.

## 4. Experimental Results and Analysis

### 4.1 Experimental Environment Configuration

The algorithm research and network training in this article are all carried out in the laboratory server. The specific computer system and experimental environment configuration used are shown in Table 2.

**TABLE 2:** Experimental environment configuration.

Project	Configuration
Operating system	Windows10
CPU	i7-9700k
GPU	RTX2080 Ti
RAM	DDR5 16 GB × 4
Programming language	Python
Deep learning framework	PyTorch

Based on the good ecology and scalability of the Python language and the open source framework PyTorch, this article uses a series of open source libraries and toolkits to implement the overall algorithm program (Neuhauser et al., 2020; Sun et al., 2020). Such as: Numpy, Albumentations, segmentation_models.pytorch semantic segmentation model library, etc.

These open source tools greatly save the development time of the defect detection and segmentation program in this article, so that more time and energy can be invested in the research, improvement and experiment of the algorithm.

### 4.2 Model Training

#### 4.2.1 Hyperparameter Setting

Before model training, some parameters cannot be learned from data and need to be set in advance, which are hyperparameters. The setting of super parameters will directly affect the training process and the final performance of the model. In general, it is necessary to optimize the hyperparameters and select a group of optimal hyperparameters for the model network to improve the performance and effect of learning (Chen et al., 2021c; Liu et al., 2021e; 2021f; 2022).

At the same time, under certain conditions, the larger the batchsize, the better the training effect. Gradient accumulation realizes the disguised expansion of batchsize. The setting of this paper is accumulation_steps = 8.

DF-ResNeSt50 is divided into two versions, DF-ResNeSt50-V1 and DF-ResNeSt50-V2. The DF-ResNeSt50-V2 version is an improvement and optimization based on the network structure of the V1 version, from post-mask processing, data enhancement, hyperparameters, etc. to improvements and optimizations. The different settings are shown in Table 3.

**TABLE 3:** Hyperparameter setting.

Hyperparameter	Set up—V1	Set up—V2
Size of the picture	256 × 1600	256 × 1600
Batch_size	4	4
Num_workers	4	6
Initial learning rate	0.01	0.005
Accumulation_steps	8	8

Except for DF-ResNeSt50-V2, the hyperparameters of other networks are all trained in accordance with Set up—V1, in Table 3.

This paper adopts ADAM optimizer to optimize learning rate and gradient descent in time. The evaluation indexes such as BCEwithLogitsloss, mIOU and DICE are introduced to evaluate the network.

#### 4.2.2 Comparison of Training Results

The experiment trained a total of 6 network models: PSP (ResNeSt14), Unet (ResNeSt14), FPN (ResNet50), FPN (ResNeSt50), DF-ResNeSt50-V1, DF-ResNeSt50-V2. Each network is trained for 40 epochs (rounds), each epoch is about 17–22 min, and each network is trained for about 12–15 h.

The detection results of steel plate defects by different networks are shown in Table 4.

**TABLE 4:** Comparison of training results.

Network	IoU (%)	mIoU (%)	Dice (%)
PSP(ResNeSt14)	63.97	54.70	78.03
Unet(ResNeSt14)	67.69	56.95	80.73
FPN(ResNeSt14)	68.92	58.35	81.60
FPN(ResNeSt50)	72.18	61.49	83.84
DF-ResNeSt50-V1	**75.89**	**65.03**	**86.29**
DF-ResNeSt50-V2	**77.15**	**65.87**	**87.10**

Bold values indicates the highest values.

**FIGURE 8:**
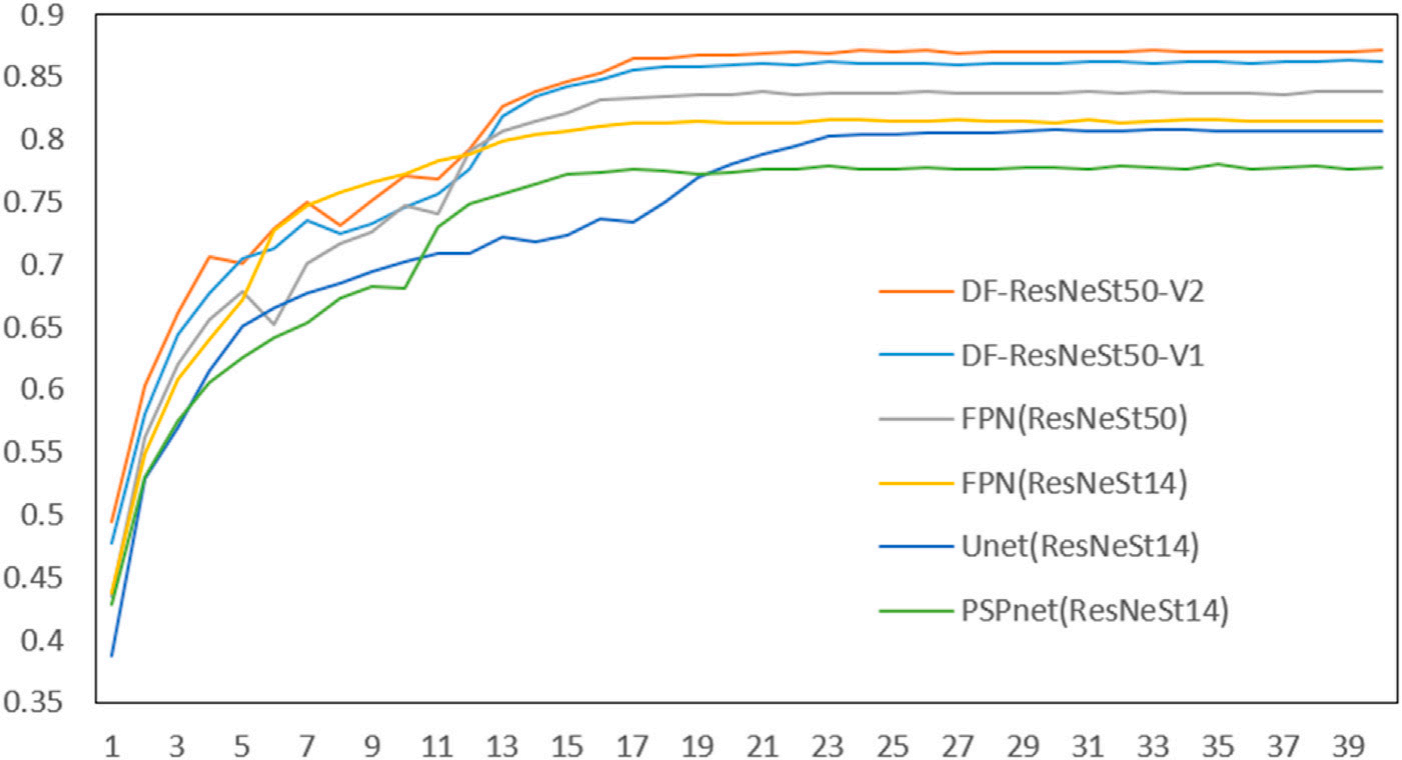
Comparison of different network models.

As shown in Table 4, among the 6 network models, the best network model is DF-ResNeSt50-V2. And, the Dice comparison of different network models is shown in Figure 8.

**FIGURE 9:**
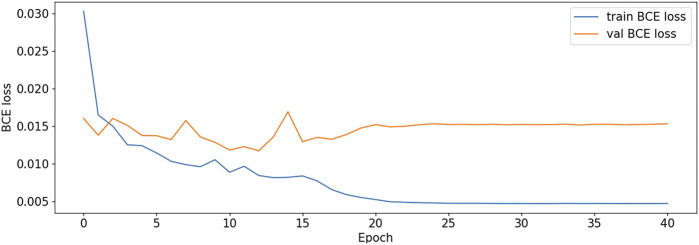
BCE Loss plot-V1.

**FIGURE 10:**
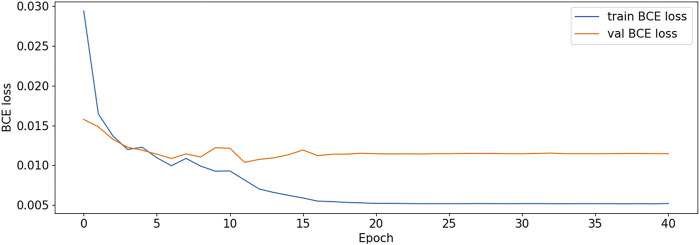
BCE Loss plot-V2.

**FIGURE 11:**
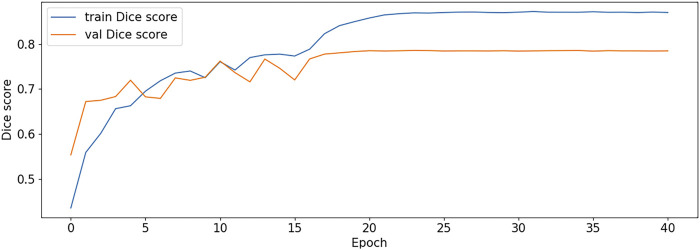
Dice score plot-V1.

**FIGURE 12:**
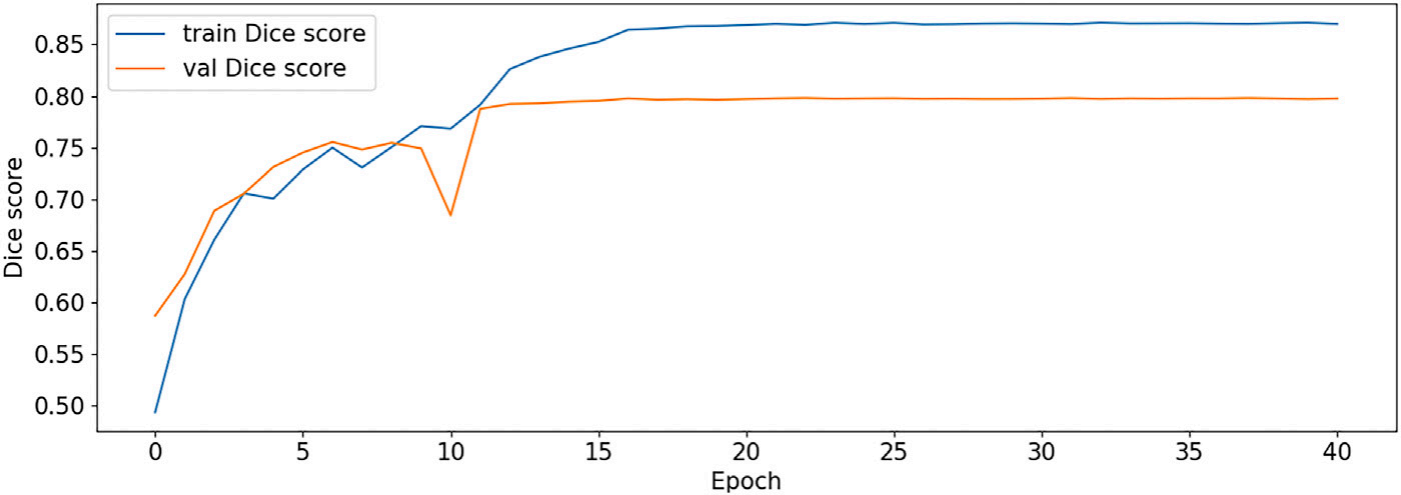
Dice score plot-V2.

**FIGURE 13:**
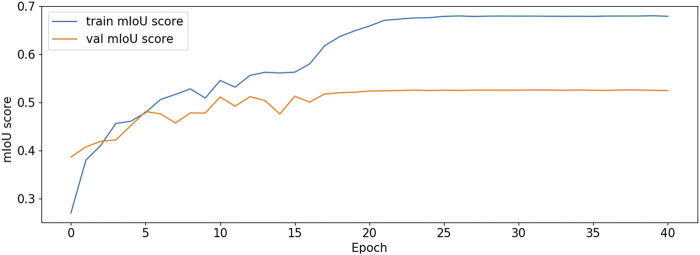
mIoU plot-V1.

**FIGURE 14:**
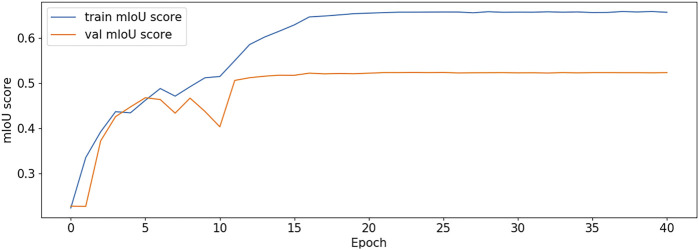
mIoU plot-V2.

The BCE Loss, Dice and mIoU of DF-ResNeSt50-V1 and V2 are shown in Figures 9–14 respectively.

DF-ResNeSt50-V2 is in the 10th epoch reducing learning rate of group 0–1.00e-04. Therefore, a mutation occurred in the 10th epoch. In the training, we use the ReduceLROnPlateau method, BCEwithLogitsloss optimizer and loss function, so this mutation is normal.

**FIGURE 15:**
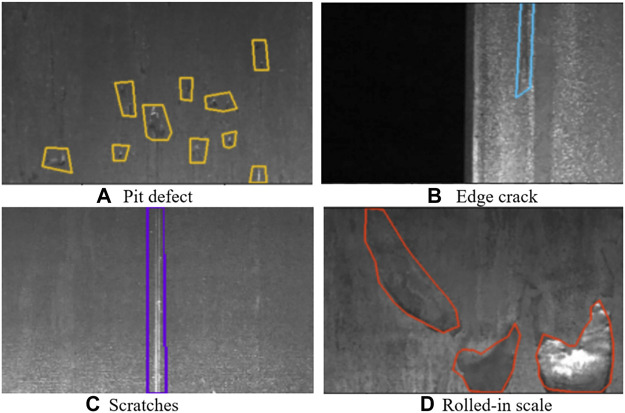
Defect detection segmentation effect. **(A)** Pit defect **(B)** Edge crack, **(C)** Scratches **(D)** Rolled-in scale.

In order to test the capability and effectiveness of the model, the effect of defect segmentation on the surface of the steel plate was visualised, with different defect types selected by different colour boxes. Figure 15 shows the visualisation of the defects in Figure 1.

After a series of network improvements and algorithm optimisation, the DF model in this paper achieves a mIOU of 77.15% and a DICE of 87.10%. It better meets the needs of defect detection in the actual steel production process and provides help for the next intelligent and efficient detection of defects.

## 5. Conclusion

In order to solve the problem of steel defects with different sizes, low contrast and different defect categories, this paper uses the DF-ResNeSt50 network model to investigate steel defects. By analyzing the surface defect data of the steel plate, the data is pre-processed with ColorJitter, Random VerticalFlip, Normalize, etc. Based on the visual attention mechanism in the bionic algorithm, this paper combined with feature pyramid networks and split attention network model, from the perspectives of data enhancement, multi-scale feature fusion and network structure optimization, etc., the DF-ResNeSt50 network model is proposed. DF-ResNeSt50 uses the radix-major implementation block (cardinality = 2, radix = 4, width of bottleneck = 40), which has better detection performance and detection efficiency compared with related networks. In the future, the correlation optimisation of the network can be applied in the direction of object detection, video detection, quality detection, scene semantic understanding, etc., with broad application prospects.

## Data Availability

The raw data supporting the conclusion of this article will be made available by the authors, without undue reservation.
